# Aging‐related prognosis analysis of definitive radiotherapy for very elderly esophageal cancer

**DOI:** 10.1002/cam4.1456

**Published:** 2018-04-02

**Authors:** Yong‐Chun Zhou, Li‐Li Chen, Hong‐Bo Xu, Qian Sun, Qi Zhang, Han‐Fei Cai, Hao Jiang

**Affiliations:** ^1^ Department of Radiation Oncology The First Affiliated Hospital of Bengbu Medical College No. 287, Chang Huai Road Bengbu 233000 China

**Keywords:** Concurrent chemoradiation, definitive radiotherapy, neutrophil–lymphocyte ratio, nutritional risk index, very elderly esophageal cancer

## Abstract

Because of the exclusion for the patients more than 75 years (very elderly patients) in many clinical trials of esophageal cancer (EC), there is no consensus on prognosis and treatment for this population. We aim to evaluate the outcomes and aging‐related prognostic factors of definitive radiotherapy (RT) for very elderly EC patients. We retrospectively analyzed 149 very elderly EC patients consecutively treated between January 2015 and June 2016 by definitive intensity‐modulated radiotherapy (IMRT) with or without chemotherapy. The clinical outcome and toxicities were assessed, and the potential prognostic factors, such as nutritional risk index (NRI) and neutrophil–lymphocyte ratio (NLR), were analyzed statistically. The median follow‐up time for survivors was 22.5 months. The 2‐year overall survival (OS), local–regional failure‐free survival (LRFFS), and distant metastasis‐free survival (DMFS) were 51.6%, 54.7%, and 85.2%, respectively. Independent predictors for poorer OS were higher American Joint Committee on Cancer (AJCC) stage, lower NRI, and higher NLR value before RT. Meanwhile, the total dose (cutoff value 60 Gy) of planning gross tumor volume (PGTV) and chemotherapy was also identified as independent prognostic indicator for LRFFS and DMFS, respectively. 72 patients had treatment failure and 58 (80.6%), 6 (8.3%), and 18 (25.0%) patients had experienced local, regional, and distant failure, respectively. Few severe toxicities were observed. The conservative definitive RT with modern technique was effective for very elderly EC patients in short term with low rate and tolerable toxicities. Local residue or recurrence was the most common failure pattern. The aging‐related prognostic factors concerned nutrition and immune, such as NRI and NLR before RT, should be considered for use in future clinical practice.

## Introduction

Based on the latest report in 2017 1, 2, esophageal cancer (EC) was the sixth most common cancer and the fourth most leading cause of cancer death in China 2013. With the increasing of life expectancy and the aging of the population, the number of elderly EC patients has grown in recent decades. According to the estimated data of 2015 in China, the patients more than 75 years (which will be called “very elderly patients” below) accounted for approximately 20% in the distribution of EC morbidity, which also had a higher mortality by 31.1% 3. Therefore, the clinical strategy related with this population should be closely concerned.

Esophagectomy is considered as the standard treatment for resectable patients with EC 4, due to multiple comorbidities and physiological changes associated with aging 5, and very elderly EC patients were difficult to tolerate the radical surgery, which often leads the higher in‐hospital mortality and lower overall survival (OS) rate than younger patients based on the systematic review 6. Instead of surgery, conservative definitive chemoradiotherapy (CRT) or radiotherapy (RT) alone was used for many very elderly EC patients depended on the noninferiority results and lower side effects 7. Despite the progress of RT technique and the development of new drugs, the OS of very elderly EC patients was also limited, which was less than 30% in 5 years 8, 9. Therefore, some potential pretreatment prognostic factors, especially associated with aging, were worth further exploring.

Malnutrition and immunity decline, which are common conditions at diagnosis in elderly EC patients, often impaired performance status, quality of life, response to treatment, and even survival 10. Recently, some studies have reported the prognostic value of baseline nutrition and immune status for OS in EC patients with definitive CRT or surgery 11, 12, 13. However, because of inadequate number of cases in these studies, roles of nutrition and immune indexes, such as nutritional risk index (NRI) and neutrophil–lymphocyte ratio (NLR), remain to be confirmed in very elderly EC patients.

In this study, we retrospectively observed the outcomes of very elderly EC patients treated with definitive RT/CRT by modern technique, and analyzed the prognostic value of some aging‐related potential factors, such as nutrition and immune indexes.

## Materials and Methods

### Patients

A total of 149 consecutive diagnosed ≥75 years very elderly EC patients received definitive CRT/RT in the first affiliated hospital of Bengbu medical college between January 2015 and June 2016. Hematologic, imageological, and pathological examination were used for pretreatment evaluations regularly. NRI before RT was calculated by the following formula: 1.519 × serum albumin level (g/L) + 41.7 × (weight before RT/baseline weight), and the patients with or without the risk of malnutrition were stratified by the NRI value <100 or ≥100 14. NLR before RT was calculated as the ratio of the absolute neutrophils count divided by the absolute lymphocyte count. All patients had proved esophageal squamous cell carcinoma pathologically, no distant organ metastasis at presentation, and no anticancer treatment history. Clinical stages were classified by the seventh edition of the American Joint Committee on Cancer (AJCC) staging system. The clinical characteristics are listed in Table 1.

**Table 1:** Patient and treatment characteristics of very elderly esophageal cancer (EC)

Characteristics	No. of patients (%)	NRI before RT		*P*	NLR before RT		*P*
		<100	≥100		<2.5	≥2.5	
Age (years) (median, range)	79 (75–91)						
<80	82 (55.0%)	31	51	0.078	48	34	0.684
≥80	67 (45.0%)	35	32		37	30	
Sex							
Male	97 (65.1%)	42	55	0.913	52	45	0.065
Female	52 (34.9%)	23	29		36	16	
Comorbidities							
No	86 (57.7%)	39	47	0.620	46	40	0.156
Yes	63 (42.3%)	26	37		41	22	
ECOG performance status							
0–1	142 (95.3%)	65	78	0.252	82	60	0.463
2–3	7 (4.7%)	5	2		3	4	
Primary tumor location							
Upper–middle	86 (57.7%)	38	48	0.872	51	35	0.792
Middle–lower	63 (42.3%)	27	36		36	27	
Primary tumor length (cm)							
<6	63 (42.3%)	28	35	0.696	38	25	0.614
≥6	86 (57.7%)	41	45		49	37	
AJCC stage							
I–II	64 (43.0%)	25	39	0.405	41	23	0.223
III	85 (57.0%)	39	46		46	39	
T stage							
1–2	18 (12.1%)	7	11	0.908	13	5	0.591
3–4	131 (87.9%)	52	77		75	56	
N status							
Negative	93 (62.4%)	41	52	0.781	55	40	0.782
Positive	56 (37.6%)	26	30		30	24	
Range of CTV							
IFI	124 (83.2%)	55	69	0.738	72	52	0.849
ENI	25 (16.8%)	12	13		14	11	
Fraction dose (Gy)							
<2	32 (21.5%)	18	14	0.203	23	9	0.096
≥2	117 (78.5%)	51	66		65	52	
Total dose for PGTV (Gy)							
<60	26 (17.4%)	14	12	0.355	17	9	0.470
≥60	123 (82.6%)	54	69		71	52	
Total dose for PTV (Gy)							
≤50	32 (21.5%)	15	17	0.874	18	14	0.918
>50	117 (78.5%)	53	64		67	50	
Chemotherapy							
No	85 (57.0%)	39	46	0.796	47	38	0.618
Yes	64 (43.0%)	28	36		38	26	

AJCC, American Joint Committee on Cancer; ECOG, Eastern Cooperative Oncology Group; ENI, elective nodal irradiation; IFI, involved field irradiation; NRI, nutritional risk index; NLR, neutrophil–lymphocyte ratio; PGTV, planning gross tumor volume; PTV: planning target volume; RT, radiotherapy.

This study was conducted in accordance with the ethical standards of the World Medical Association Declaration of Helsinki and was approved by the ethics committee of the first affiliated hospital of Bengbu Medical College.

### Radiotherapy

All patients were treated with intensity‐modulated radiotherapy (IMRT) by simultaneous integrated boost (SIB) approach. For RT field, the gross tumor volume (GTV) was defined as visible esophageal primary tumor (GTV‐T) and imageological positive lymph nodes (GTV‐N) shown on localizable computed tomography (CT), which also referred to endoscopy, barium swallow, positron emission tomography (PET), and scale of organ mobility. For involved field irradiation (IFI), the clinical target volume (CTV) consisted of CTV‐T and involved lymph nodes. The CTV‐T was defined as the GTV‐T plus 3 cm cranial–caudal and 0.7 cm radial margin. The planning target volume (PTV) was generated by additional 0.5 cm radial margin for CTV‐T and 1 cm radial margin for GTV‐N. For elective nodal irradiation (ENI), besides CTV‐T, the CTV also include extended lymphatic drainage area at risk, which referred to the regions of primary tumor and positive lymph node, and the PTV was created 0.5 cm radial margin from GTV or CTV, which were named PGTV‐T, PGTV‐N, and PTV. The prescribed doses were 50–66 Gy for both PGTV‐T and PGTV‐N in 25–35 fractions and 45–57.6 Gy for PTV, respectively.

### Chemotherapy

64 patients underwent chemotherapy in different phases during treatment, which including concurrent chemoradiation (CCRT) for 36 patients, neoadjuvant chemotherapy for 14 patients, and adjuvant chemotherapy for 20 patients. The chemotherapeutic regimens were mostly based on 5‐fluorouracil (Fu); moreover, oral S‐1 or capecitabine alone was mainly used for CCRT (36/36, 100%) and adjuvant chemotherapy (13/20, 65%).

### Follow‐up

All patients were evaluated every 3 months in the first 2 years and every 6 months thereafter. The complete evaluation during follow‐up included hematologic, imageological, and pathological examination mentioned in pretreatment evaluations. The failure of treatment was assessed by pathological or imageological evidence. The residue or recurrence of the primary tumor and regional lymph nodes was defined as local and regional failure, and the metastasis to other organs or non‐regional lymph nodes was identified as distant failure. RT‐related toxicities were determined by the radiation morbidity scoring criteria of Radiation Therapy Oncology Group (RTOG).

### Statistical analysis

All radiation doses were converted into the equivalent dose in 2 Gy per fraction (EQD2) with the value of 10 for *α*/*β*. The cutoff value of NLR was determined by ROC curve. Differences in categorical variables were analyzed by chi‐squared test. OS, local–regional failure‐free survival (LRFFS), and distance metastasis‐free survival (DMFS) were identified as the time from the first date of therapy until the date of death, local–regional failure, and distant metastasis, respectively, and they were computed by Kaplan–Meier method. The univariate and multivariate analysis were operated by log‐rank test and Cox proportional hazard model, respectively, and the potential prognostic factors with *P *< 0.1 in univariate analysis were screened for multivariate analysis. *P* < 0.05 was considered statistical significance. SPSS 16.0 (SPSS, Chicago, IL) was used for statistical analysis.

## Results

### Patients’ characteristics

Patients’ characteristics are presented in Table 1. The median age was 79 years. Based on the AJCC staging system (7th edition), 85 patients were distributed in stage III. IFI was used in 124 patients, and the other 25 patients received ENI. All the patients completed the RT prescription, and the actual median radiation dose for PGTV was 62 Gy (range, 50–66 Gy).

### Patient and treatment characteristics were unrelated to NRI and NLR

According to ROC curve analysis, 2.5 was calculated as the optimal cutoff value for NLR before RT (sensitivity = 57.97%, specificity = 74.03%) (Figure S1). Each characteristic of very elderly EC patients showed no significant relationship with different NRI and NLR stratification (Table 1).

### Treatment outcomes and prognostic analysis

**Figure 1:**
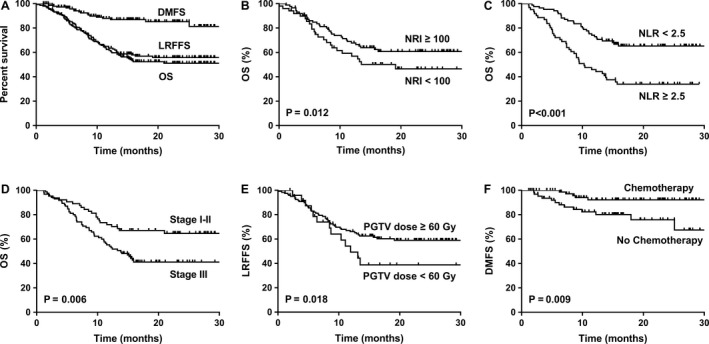
Kaplan–Meier estimates of clinical outcome for very elderly esophageal cancer (EC) patients. (A) OS, LRFFS, and DMFS for total patients. (B) OS for NRI <100 and ≥100. (C) OS for NLR <2.5 and ≥2.5. (D) OS for AJCC stage I–II and III. (E) LRFFS for PGTV dose <60 and ≥60. (F) DMFS for chemotherapy and no chemotherapy.

**Figure 2:**
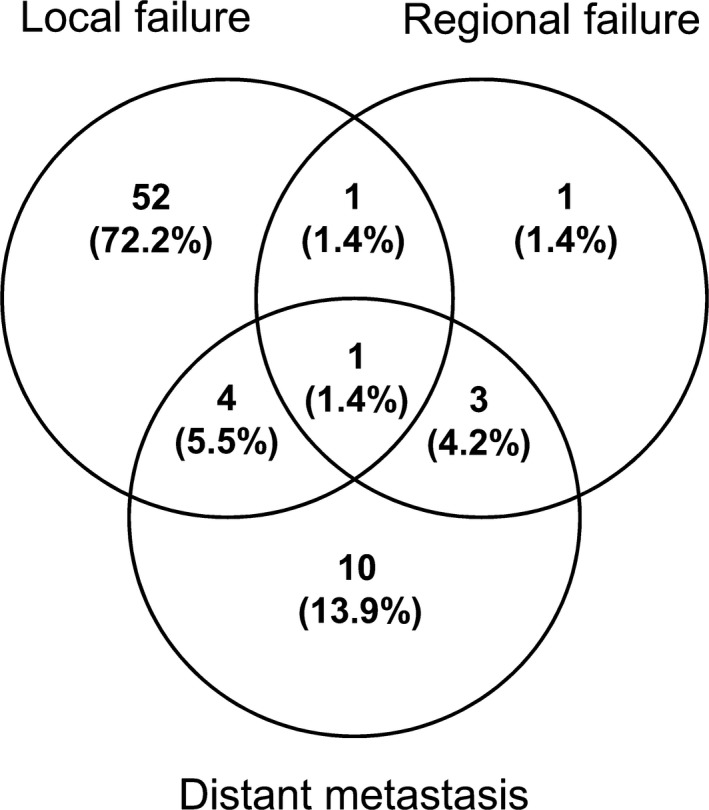
Distribution of failure patterns in very elderly EC patients.

The median follow‐up time for survivors was 22.5 months (range, 13.2–30.7 months), and the 2‐year OS, LRFFS, and DMFS were 51.6%, 54.7%, and 85.2%, respectively (Fig. 1A). At the latest time of follow‐up, 72 patients developed treatment failure, 58 (80.6%), six (8.3%), and 18 (25.0%) patients had experienced local failure, regional failure, and distant metastasis, respectively, and the details are shown in Figure 2.

In the patients with NRI ≥100 before RT had better OS (*P *=* *0.012) and DMFS (*P *=* *0.011) than the patients with NRI < 100, and NLR ≥ 2.5 before RT was associated with bad OS (*P *<* *0.001) and LRFFS (*P *=* *0.002). The results of univariate analysis for all of involved clinical characteristics are also shown in Table 2 and Figure 1.

**Table 2:** Univariate analysis of patient and treatment characteristics on clinical results for very elderly EC

Characteristics	No. of patients (%)	2‐year OS	2‐year LRFFS	2‐year DMFS
Age (years) (median, range)	79 (75–91)	*P *=* *0.373	*P *=* *0.057	*P *=* *0.068
<80	82 (55.0%)	55.2%	62.2%	92.5%
≥80	67 (45.0%)	46.6%	46.1%	80.0%
Sex		*P *=* *0.398	*P *=* *0.849	*P *=* *0.720
Male	97 (65.1%)	48.8%	54.2%	84.7%
Female	52 (34.9%)	55.8%	56.1%	86.3%
Comorbidities		*P *=* *0.438	*P *=* *0.217	*P *=* *0.517
No	86 (57.7%)	48.0%	51.0%	79.9%
Yes	63 (42.3%)	55.7%	60.1%	83.3%
ECOG performance status		*P *=* *0.565	*P *=* *0.333	*P *=* *0.364
0–1	142 (95.3%)	51.6%	55.6%	84.6%
2–3	7 (4.7%)	42.9%	34.3%	100.0%
NRI before RT		*P *=* *0.012	*P *=* *0.093	*P *=* *0.011
<100	54 (36.2%)	44.8%	50.0%	74.1%
≥100	95 (63.8%)	60.1%	63.3%	91.7%
NLR before RT		*P *<* *0.001	*P *=* *0.002	*P *=* *0.787
<2.5	87 (58.4%)	65.2%	64.1%	87.8%
≥2.5	62 (41.2%)	34.0%	39.6%	87.1%
Primary tumor location		*P *=* *0.446	*P *=* *0.454	*P *=* *0.476
Upper–middle	86 (57.7%)	48.4%	51.6%	86.7%
Middle–lower	63 (42.3%)	53.8%	57.4%	82.3%
Primary tumor length (cm)		*P *=* *0.354	*P *=* *0.672	*P *=* *0.780
<6	63 (42.3%)	45.5%	50.2%	82.1%
≥6	86 (57.7%)	55.5%	57.2%	86.9%
AJCC stage		*P *=* *0.006	*P *<* *0.001	*P *=* *0.050
I–II	64 (43.0%)	64.7%	73.8%	91.5%
III	85 (57.0%)	41.2%	39.2%	80.8%
T stage		*P *=* *0.377	*P *=* *0.226	*P *=* *0.280
1–2	18 (12.1%)	61.1%	68.8%	93.3%
3–4	131 (87.9%)	49.6%	52.6%	83.8%
N status		*P *=* *0.389	*P *=* *0.082	*P *=* *0.192
Negative	93 (62.4%)	54.6%	62.1%	87.9%
Positive	56 (37.6%)	45.9%	42.9%	80.8%
Range of CTV		*P *=* *0.640	*P *=* *0.502	*P *=* *0.819
IFI	124 (83.2%)	52.1%	55.8%	85.1%
ENI	25 (16.8%)	46.8%	50.1%	86.7%
Fraction dose (Gy)		*P *=* *0.495	*P *=* *0.654	*P *=* *0.804
<2	32 (21.5%)	45.9%	51.2%	84.1%
≥2	117 (78.5%)	52.6%	55.6%	85.6%
Total dose for PGTV (Gy)		*P *=* *0.055	*P *=* *0.018	*P *=* *0.552
<60	26 (17.4%)	32.2%	37.3%	89.3%
≥60	123 (82.6%)	55.0%	58.0%	84.4%
Total dose for PTV (Gy)		*P *=* *0.295	*P *=* *0.530	*P *=* *0.390
≤50	32 (21.5%)	45.5%	51.5%	95.5%
>50	117 (78.5%)	52.6%	54.9%	82.6%
Chemotherapy		*P *=* *0.695	*P *=* *0.310	*P *=* *0.009
No	85 (57.0%)	50.5%	50.6%	75.9%
Yes	64 (43.0%)	52.7%	51.0%	92.2%

AJCC, American Joint Committee on Cancer; DMFS, distance metastasis‐free survival; ECOG, Eastern Cooperative Oncology Group; ENI, elective nodal irradiation; IFI, involved field irradiation; LRFFS, local–regional failure‐free survival; NRI, nutritional risk index; NLR, neutrophil–lymphocyte ratio; OS, overall survival; PGTV, planning gross tumor volume; PTV: planning target volume; RT, radiotherapy.

In further multivariate analysis, NRI before RT was identified as independent prognostic indicator for OS and DMFS, NLR, and AJCC stage were both significant predictors for OS and LRFFS; moreover, PGTV dose (cutoff value 60 Gy) and chemotherapy could predict LRFFS and DMFS, respectively (Table 3).

**Table 3:** Multivariate analysis of prognostic factors on clinical results for very elderly EC

Endpoint	Prognostic factors	Multivariate analysis	
		*P*	HR (95% CI)
2‐year OS	NRI before RT (<100 vs. ≥100)	0.039	0.887 (0.360–0.972)
	NLR before RT (<2.5 vs. ≥2.5)	<0.001	2.452 (1.513–3.972)
	AJCC stage (I–II vs. III)	0.019	1.858 (1.108–3.114)
2‐year LRFFS	NLR before RT (<2.5 vs. ≥2.5)	0.006	2.053 (1.227–3.436)
	AJCC stage (I–II vs. III)	0.001	2.620 (1.453–4.723)
	PGTV dose (<60 Gy vs. ≥60 Gy)	0.045	0.891 (0.463–0.984)
2‐year DMFS	NRI before RT (<100 vs. ≥100)	0.046	0.441 (0.128–0.992)
	Chemotherapy (No vs. Yes)	0.043	3.206 (1.080–6.451)

AJCC, American Joint Committee on Cancer; DMFS, distance metastasis‐free survival; LRFFS, local–regional failure‐free survival; NLR, neutrophil–lymphocyte ratio; NRI, nutritional risk index; OS, overall survival; PGTV, planning gross tumor volume; RT, radiotherapy.

### Toxicities

The treatment‐related acute toxicities, including neutropenia leucopenia, thrombocytopenia, esophagitis, pneumonia, and gastrointestinal reaction, are shown in Table 4. The toxicities mainly distributed in grade 1 or 2, and the incidences of grade 3 were very low, and no grade 4 acute side effect and treatment‐related death happened.

**Table 4:** Treatment‐related acute toxicities in very elderly EC

Toxicities (No. of patients) (%)	Grade 0–1	Grade 2	Grade 3
Neutropenia	143 (96.0%)	4 (2.7%)	2 (1.3%)
Thrombocytopenia	148 (99.4%)	1 (0.6%)	0 (0.0%)
Esophagitis	129 (86.6%)	19 (12.8%)	1 (0.6%)
Pneumonia	144 (96.6%)	4 (2.7%)	1 (0.6%)
Gastrointestinal reaction	144 (96.6%)	5 (3.4%)	0 (0.0%)

## Discussion

As a noninvasive method of esophageal cancer, depending on the noninferiority clinical outcome and lower toxicity compared with surgery in retrospective data 7, 8, 9, radical RT or CRT has been adopted by more and more medical institutions for elderly patients. In this study, the reported 2‐year OS was more than 50%, and the rates of severe toxicity‐related treatment were also very low (less than 2%) in very elderly patients, which provided the feasibility of nonsurgical method. Therefore, conservative definitive RT or CRT should be an effective way for very elderly EC patients with limited life expectancy.

The relationship between RT dose and EC prognosis had been attracted more and more attention in clinic. According to the results of INT‐0123 trial 15, dose escalation could not bring survival benefits for EC patients, and 50.4 Gy was still recommended as standard dose in definitive RT by NCCN guideline. However, the previous data were mainly based on the background of conventional two‐dimension (2D) RT techniques with more severe toxicities and even treatment‐related death. Recently, as an important representative of modern RT technique, IMRT has been widely used in clinical practice with superior target volume conformality and lower dose to normal structures in physics 16. Meanwhile, the results from propensity score and meta‐analysis both showed survival advantage of IMRT for EC patients compared with traditional RT technique, such as 3D conformal RT (3D‐CRT) 17, 18. Furthermore, on the basis of IMRT, definitive RT dose ≥60 Gy has yielded more favorable survival outcomes compared with standard dose 50.4 Gy in some latest researches 19, 20, and the similar effect could be found in the elderly population without increasing treatment‐related toxicities 21. In the present study, all of very elderly patients were experienced IMRT and gained good short‐term clinical effect, moreover, the subgroup of PGTV dose ≥ 60 Gy had superior LRFFS and better trends for OS (univariate analysis *P *=* *0.055), these results indicated that dose escalation for tumor probably improve OS by enhancing LRFFS in very elderly EC patients, which also need to be further verified in prospective clinical trials.

In 3D RT period, IFI has been widely used for EC patients in clinic, but the RT fields for CTV were still controversial between ENI and IFI. The recent meta‐analysis revealed that IFI was not inferior to ENI in OS and LRFFS, even though the tumor located in cervical and upper thoracic esophagus 22, meanwhile, IFI also decreased the incidences of lung and esophagus toxicities with less RT volume compared with ENI 23. Similar results were reported for ≥70 years elderly patients in a single institute retrospective analysis, which presented noninferior effects and lower radiation esophagitis rate in IFI group 24. In our study, there was also no significant difference in survival outcome between ENI and IFI, which was consistent with many previous studies. Therefore, IFI may be a better choice to prolong survival as well as improve quality of life for very elderly EC patients compared with ENI.

CCRT has been identified as the standard treatment for inoperable EC patients as the results of landmark RTOG 8501 published, which depend on the advantage in 27% enhancement of 5‐year OS compared with RT alone 25. However, the recommended concurrent chemotherapy was based on platinum or 5‐FU double agents, how to balance the efficiency and toxicities among the kind, number, and dose of these agents for elderly EC patients, it remains unclear. Our previous study reported that oral single‐agent CCRT had no significant difference in survival outcome and lower toxicities compared with double agents CCRT 7. In our present study, although there is no direct evidence about the effects of CCRT and the difference of agents for very elderly EC patients, the improvement of DMFS benefit from chemotherapy (the 5‐FU‐based single‐agent CCRT was dominant) was showed. So, mild chemotherapy regimens may be more suitable for very elderly EC patients and also need to be verified in the future.

Because of the exclusion for the patients more than 75 years in many clinical trials, besides some prognostic factors related to the tumor characteristics and treatment information, such as stage, and chemotherapy, some other potential factors need to be identified for elderly EC patients. Although age itself was not demonstrated as risk factor for EC in previous studies 26, which was same as our studies, some factors related aging may play important role to predict prognosis. The poor prognosis or tumor progression of EC was associated with not only tumor cells themselves but also the tumor microenvironment, which partly regulated by host nutrition and immunity status 27. Malnutrition and immune inhibition occupied certain proportion of elderly EC patients, and they should be concerned at diagnosis. Recently, the nutrition and immune indexes, and pretreatment NRI and NLR had been identified as independent prognostic factors for EC patients 11, 12, 13, but they remain inconclusive for specific elderly patients. In our study, NRI and NLR before RT were both significantly correlated with patient survival, which was consistent with the studies for the all age population. However, whether the changes of these indexes values during the therapy make influence on clinical outcomes such as absolute lymphocyte count nadir 28 remains need to be clarified in the future.

There were some limitations in this study. First, this was a single institutional, retrospective study without enough follow‐up times and more exact information such as stage. Furthermore, some potential prognostic factors, such as CCRT, were not involved in this study due to some potential confounding factors and small sample size. Therefore, well‐designed prospective researches need to be conducted to further explore the potential prognostic factors in more detail.

## Conclusions

The conservative definitive RT with modern technique was effective for very elderly EC patients in short term with low and tolerable toxicities. Local residue or recurrence was the most common failure pattern. The aging‐related prognostic factors concerned nutrition and immune, such as NRI and NLR before RT, should be considered for use in future clinical practice.

## Conflicts of Interest

The authors declare that they have no conflict of interests.

## Supporting information


**Figure S1.** ROC for NLR.
