# Supplementary material for: Aging‐related prognosis analysis of definitive radiotherapy for very elderly esophageal cancer
Source: Cancer Med. 2018 Apr 2;7(5):1837–44. doi: 10.1002/cam4.1456 (PMC5943545; doi:10.1002/cam4.1456)
Supplement: Supplementary file 1 — Figure S1. ROC for NLR. [file CAM4-7-1837-s001.docx]

ROC for NLR


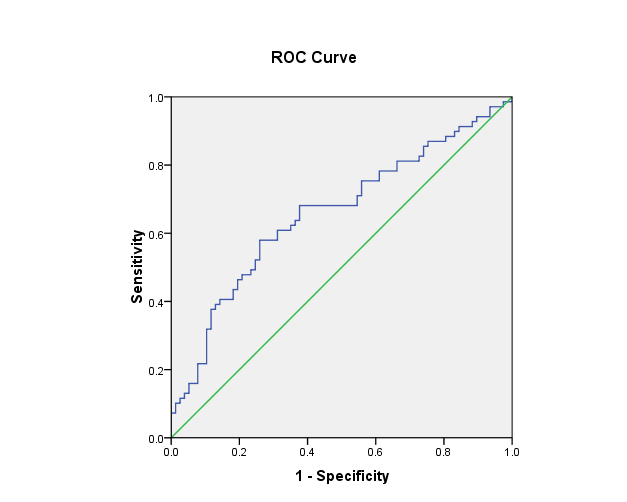


| **Area Under the Curve** | | | | |
| --- | --- | --- | --- | --- |
| Test Result Variable(s):NL | | |  |  |
| Area | Std. Error^a^ | Asymptotic Sig.^b^ | Asymptotic 95% Confidence Interval | |
|  |  |  | Lower Bound | Upper Bound |
| .659 | .046 | .001 | .569 | .749 |
| a. Under the nonparametric assumption | | | |  |
| b. Null hypothesis: true area = 0.5 | | |  |  |
